# Supplementary material for: NMK-TD-100, a Novel Microtubule Modulating Agent, Blocks Mitosis and Induces Apoptosis in HeLa Cells by Binding to Tubulin
Source: PLoS One. 2013 Oct 7;8(10):e76286. doi: 10.1371/journal.pone.0076286 (PMC3792137; doi:10.1371/journal.pone.0076286)
Supplement: Table S1 — Binding Parameters. (DOC) [file pone.0076286.s001.doc]

NMK-TD-100, a Novel Microtubule Modulating Agent, Blocks Mitosis and Induces Apoptosis in HeLa Cells by Binding to Tubulin.

Surela Bhattacharya^1^, N Maruthi Kumar^2^, Arnab Ganguli^1^, Mukund P Tantak^2^, Dalip Kumar*^2^ and Gopal Chakrabarti*^1^

^1^Department of Biotechnology and Dr. B.C. Guha Centre for Genetic Engineering and Biotechnology, University of Calcutta, 35 Ballygunge Circular Road Kolkata, WB 700019, India. ^2^Department of Chemistry, Birla Institute of Technology and Science, Pilani, Rajasthan 333031, India.

*Correspondence to:

Gopal Chakrabarti

Department of Biotechnology and Dr. B.C. Guha Centre for Genetic Engineering and Biotechnology, University of Calcutta.

35 Ballygunge Circular Road, Kolkata, WB 700 019. India.

Telephone: 91-33-2461-5445; Fax : 91-33-2461-4849

Email : gcbcg@caluniv.ac.in

Or

Dalip Kumar

Department of Chemistry, Birla Institute of Technology and Science,

Pilani, Rajasthan 333031, India.

Telephone: 91-1596-24507; Fax : 91-1596-244183

Email: [**dalipk@pilani.bits-pilani.ac.in**](mailto:dalipk@pilani.bits-pilani.ac.in).

Short Title - NMK-TD-100 binds tubulin and induces apoptosis.

Abstract: Thiadiazoles are one of the most widely utilized agents in medicinal chemistry, having a wide range of pharmacologic activity. Microtubules (MTs) have always remained a sought-after target in rapidly proliferating cancer cells. We screened for the growth inhibitory effect of synthetic 5-(3-indolyl)-2-substituted-1,3,4-thiadiazoles on cancer cells and identified NMK-TD-100, as the most potent agent. Cell viability experiments using human cervical carcinoma cell line (HeLa cells) indicated that the IC_50_ value was 1.42±0.11 µM for NMK-TD-100 for 48 h treatment. In further study, we examined the mode of interaction of NMK-TD-100 with tubulin and unraveled the cellular mechanism responsible for its anti-tumor activity. NMK-TD-100 induced arrest in mitotic phase of cell cycle, caused decline in mitochondrial membrane potential and induced apoptosis in HeLa cells. Immunofluorescence studies using an anti-α-tubulin antibody showed a significant depolymerization of the interphase microtubule network and spindle microtubule in HeLa cells in a concentration-dependent manner. However, the cytotoxicity of NMK-TD-100 towards human peripheral blood mononuclear cells (PBMC) was lower compared to that in cancer cells. Polymerization of tissue purified tubulin into microtubules was inhibited by NMK-TD-100 with an IC_50_ value of 17.5±0.35 μM. The binding of NMK-TD-100 with tubulin was studied using NMK-TD-100 fluorescence enhancement and intrinsic tryptophan fluorescence of tubulin. The stoichiometry of NMK-TD-100 binding to tubulin is 1:1 (molar ratio) with a dissociation constant of ~1 µM. Fluorescence spectroscopic and molecular modeling data showed that NMK-TD-100 binds to tubulin at a site which is very near to the colchicine binding site. The binding of NMK-TD-100 to tubulin was estimated to be ~10 times faster than that of colchicine. The results indicated that NMK-TD-100 exerted anti-proliferative activity by disrupting microtubule functions through tubulin binding and provided insights into its potential of being a chemotherapeutic agent.

Key Words:

Cervical cancer; tubulin-microtubule equilibrium; synthetic thiadiazole compound; antimicrotubule agent; apoptosis; fluorescence spectroscopy.

Introduction

Worldwide, cervical cancer is considered to be the second most common form of cancer as far as mortality and incidence are concerned and India contributes to about 20–30% of the global burden [1]. Cervical cancer is the most common malignancy among Indian women. In developed countries, the widespread use of cervical screening program has dramatically reduced the incidence of invasive cervical cancer [2]. In contrast, over a span of 25-year, the number of cases of cervical cancer has steadily increased in India, with over 80% of cases occurring amongst rural women. The treatment of cervical cancer varies with the stages of development of the cancer. Early stage cancers can be eradicated by surgery and radiation therapy. Advanced stage tumors are treated with radiation therapy and cisplatin-based chemotherapy. In 2006, the US Food and Drug Administration approved the use of a combination of two chemotherapy drugs, hycamtin and cisplatin for women with late-stage cervical cancer treatment [3]. However, combination treatment has significant risk of neutropenia, anemia, and thrombocytopenia side effects. Therefore, there is always a quest for new chemotherapeutic agents which will be effective in killing the cervical cancer cells with minimal toxicity to the subject.

Microtubules are cytoskeletal hollow fibers present in most eukaryotic cells, are among the most successful targets for anticancer therapeutics [4]. These dynamic structures result from the interaction of α/β tubulin polymers with microtubule-associated proteins (MAPs) [5]. Microtubules perform various functions in cells such as maintenance of cell shape and processes such as motility, mitosis, intracellular vesicle transport, organization, and positioning of membranous organelles [6]. Microtubule-targeted agents inhibit mitosis in the rapidly dividing cancer cells by interfering with the dynamics of the spindle microtubules, which are required for normal mitotic progression [7]. Microtubule-targeted anti-mitotic compounds are usually classified into two main groups based on their mode of action [8]. One group, known as microtubule-destabilizing agents, inhibits microtubule polymerization and promotes microtubule depolymerization, such as *vinca* alkaloids, colchicines, podophyllotoxin and nocodazole. The second group characterized as microtubule-stabilizing agents, inhibits microtubule depolymerization and stabilizes microtubules. The second group constitutes of paclitaxel, epothilones, discodermolide, laulilamide and many more. The anti-microtubule agents affect microtubule-polymer mass as well as their dynamics. In spite of structural diversity among the antimicrotubule agents, often they employ a common mechanism of action. Taxanes [9], vinca alkaloids [10], vitamin K3 [11] and many other ligands have been reported to exert favorable effects in cervical cancer. However, resistance to anti-microtubule agents, particularly during multiple cycles of therapy [12] and their toxicity and other side effects on human physiology have always prompted the researchers in identifying and developing novel anti-microtubule agents.

Recent development in pharmaceutical science has led the path to the discovery of small molecules as effective anti-cancer agents [13]. A wide range of heterocyclic ring systems has been studied for the development of novel chemical entities as a lead molecule in the drug discovery procedure [14]. Thiadiazoles are one of the well-known structural fragments in medicinal chemistry having broad spectrum of pharmacological activities [15]. Particularly, 1,3,4-thiadiazoles are much explored for their broad spectrum of biological activities including anti-inflammatory [16], antihypertensive [17], antibacterial [18], anticonvulsant, antimicrobial [19], antidepressants [20], anti-leishmanial [21] and anticancer [22, 23]. Furthermore, widely explored 2-aminothiadiazoles are in clinical trials for the treatment of patients with different cancer types [24]. Among the important heterocycles, many of the natural and synthetic indole-based heterocycles with diverse mechanism of action have been reported as lead anticancer molecules [25]. Various indolyl azoles and bisindolylazoles are known for their anticancer activities. Camalexin (indolylthiazole) which is a phytoalexin was detected and isolated from the leaves of *Cruciferae Camelina sativa* infected with *Alternaria brassicae*. Analogues of camalexin were evaluated for their cytotoxic activity against human breast tumor cell lines. A thiazolyl indolequinone, BE 109881b isolated from culture broths of Streptomyces strain, is known to increase DNA-topoisomerase complex formation and displayed significant anticancer activities [26]. Promising activities of natural thiadiazoles prompted us to study the potential of 5-(3-indolyl)-2-substituted-1,3,4-thiadiazoles as anti mitotic agents. The cytotoxic status of these 5-(3-indolyl)-2-substituted-1,3,4-thiadiazoles has already been reported in some cancer cell lines [14] and this study acquired our attention to unveil the possible antimitotic role of the most effective compound. The cytotoxicity of the compounds in HeLa cells revealed that NMK-TD-100 was the most prominent one in killing those cancer cells. Further studies were done to elucidate the mechanism of action of NMK-TD-100 in both cellular and *in cell-free system*. The results of the study demonstrated that NMK-TD-100 depolymerized the microtubule, caused G2/M arrest and subsequently caused apoptosis in HeLa cells. *In cell free system* it also inhibited polymerization of tubulin into MTs and it bound tubulin near colchicine binding site with high affinity.

Materials and Methods:

Ethics Statement: In this works although goat brains were used for purification of protein. In India goat brain is commercially available in the butcher shop and we purchased fresh brain tissues form the market (Hazi MD. Yaqub & Sons Meat Shop, Shop No. 786, Kolkata, India) and purified tubulin. Permission was obtained from this butcher shop to use this animal part for protein purification. No animal ethics committee approval is required since selling goat brains is not illegal in India.

The collection of human blood from adult healthy volunteers and subsequent experiments with Human Peripheral Blood mononuclear (PBMC) cells were approved by the Institutional Bioethics Committee for animal and human research studies, University of Calcutta, following the Code of Ethics of the World Medical Association (Declaration of Helsinki) for experiments involving humans.

Materials

Nutrient mixture Dulbecco’s minimal essential medium (supplemented with 1 mM L-glutamine), fetal bovine serum, penicillin-streptomycin, amphotericin B and 0.25% Trypsin-EDTA were purchased from GIBCO (Invitrogen), USA. Colchicine, vinblastine and DAPI were purchased from Sigma, USA. Mouse monoclonal anti-α-tubulin antibody, mouse monoclonal anti-p53 antibody, rabbit polyclonal anti-Bcl-2 antibody, goat polyclonal caspase-3 antibody, mouse monoclonal anti-bax antibody and annexin V-FITC apoptosis kit were from Santa Cruz Biotechnology (Santa Cruz, CA, USA). The Bradford protein estimation kit, goat anti-mouse IgG-HRP, rabbit anti-goat IgG-HRP, goat anti-rabbit IgG-HRP and goat anti-mouse IgG-TRITC conjugate were purchased from Genei, India. PIPES, guanosine 5'-triphosphate (GTP), MgCl_2_, L-glutamic acid and EGTA were purchased from Sisco Research Laboratories, India. All other chemicals and reagents were of analytical grade and were purchased from Sisco Research Laboratories, India. Goat brains were purchased from butcher shop.

Purification of Tubulin from Goat Brain

Tubulin was isolated from goat brain (From Bucher Shop named Hazi MD. Yaqub & Sons Meat Shop, Shop No 786, Kolkata, India) via two cycles of temperature-dependent assembly and disassembly in PEM buffer containing 50 mM PIPES, 1 mM EGTA, and 1 mM MgSO4 (pH 6.9) in the presence of 1 mM GTP, followed by two more cycles in 1M glutamate buffer, pH 6.9 [27]. Aliquots of purified tubulin were frozen in liquid nitrogen and stored at -70°C. The concentration of protein was estimated following the method of Bradford where bovine serum albumin was used as the standard [28]. All experiments were performed in PEM buffer, unless otherwise stated.

Preparation of Ligand Solution

NMK-TD compounds (Fig. 1A for structure) were synthesized according to the previous report [14]. The stock solution was prepared by weighing 1 mg of desired ligand in Sartorius CPA225D weighing balance and dissolving it in 100% DMSO. In all the subsequent experiments the effective DMSO concentration was kept below 1%. The wave-length spectrum of 100 µM NMK-TD-100 in PEM buffer is presented in Fig. 1B.

Cell Culture

Human cervical carcinoma cell line HeLa was obtained from cell repository of National Centre for Cell Science, (NCCS) Pune, India. NCCS characterized the cells by mt-rDNA sequence to confirm the species. This cell line was found to be free of mycoplasma. Cell line authentication was done by short tandem repeat profiling and isoenzyme analysis by the supplier and was also reported negative for the presence of mycoplasma. HeLa cells were cultured in nutrient mixture DMEM supplemented with 1mM L-glutamine, 10% fetal bovine serum, 3.7 gm/l NaHCO_3_, 50 μ*g*/mL penicillin, 50 μg/mL streptomycin, and 2.5 μg/mL amphotericin B. Cells were maintained at 37°C in a humidified atmosphere containing 5% CO_2_. Cells were grown to confluency of about 80% in tissue culture flasks, then trypsinized with 0.25% trypsin-EDTA and divided in subsequent culture plates as required. The morphology of normal and treated cells was kept under observation with an Olympus model CKX41 inverted microscope.

Cell Viability Assay (MTT Assay)

Cultured cells were seeded in 96 well plate at a density of 1X10^4^cells/ml and grown overnight. Cells were treated with varying concentration of NMK-TD-100 and incubated for 48 h. MTT (5 mg/ml) solution was prepared in PBS and filtered. 20 µl of MTT solution was added to each well and incubated for 4 h at 37°C. Subsequently, purple formazan crystal was dissolved in 150 µl of DMSO, added to each well and incubated for 2 h in dark at room temperature. The absorbance was measured on an ELISA reader (MultiskanEX, Lab systems, Helsinki, Finland) at a test wavelength of 570 nm and a reference wavelength of 750 nm. Data were calculated as the percentage of inhibition by the following formula:

…………………………… (1)

where A_t_ and A_s_ are the absorbance of the test substances and solvent control, respectively **[29].**

Isolation of Human Peripheral Blood mononuclear cells (PBMC) and MTT assay

Whole peripheral blood was collected from adult healthy volunteers with prior written consent. All subjects were not taking any type of medication. The PBMC isolation was made by the difference of gradient density Ficoll- Hypaque (Histopaque 1077, Sigma–Aldrich-USA) [30]. Whole blood was carefully layered on the top of equal volume of Ficoll- Hypaque. After centrifugation (400 × g, 30 min at room temperature), the PBMC were found at the plasma/ Ficoll- Hypaque interphase and collected carefully with a Pasteur pipette. After that, the cells were washed in PBS twice (240 × g for 10 min), and resuspended in RPMI 1640 medium containing 4.5 g/L glucose supplemented with 2 mM l-glutamine, penicillin/streptomycin (50 IU/mL and 50 µg/mL, respectively) and 10% (v/v) fetal bovine serum (FBS). Cytotoxicity of NMK-TD-100 using PBMC was performed by MTT assay after 48 h of treatment.

Colony Formation Assay

Cultured HeLa cells were seeded in a 6 well plate at a density of 1000 cells /well. The cells were allowed to attach to the substratum for 24 h and treated with 0, 2.5, 5 and 10 µM of NMK-TD-100 for 48 h. The cells were then washed with PBS and fresh culture media without NMK-TD-100 added to each well. The cells were allowed to grow for 10 days in normal culture condition. Then the colonies were fixed with 4% glutaraldehyde and stainded with 0.5% crystal violet solution. Excess stain was washed out and the wells were air dried. The photographs of the colonies were taken. Colony formation was quantitated by dissolving stained cells in Sorenson’s buffer (0.1 mol/L sodium citrate, 50% ethanol, pH 4.2) for colorimetric reading of OD at 550 nm [31].

Apoptosis Assay

Different concentration of NMK-TD-100 were added to cultured HeLa cells and incubated for 36 h. Approximately 1x10^5^ cells were then stained for 15 min at room temperature in the dark with fluorescein isothiocyanate (FITC)-conjugated annexin V (1 µg/ml) and propidium iodide (PI) (0.5 µg/ml) in a Ca^2+^-enriched binding buffer, and analyzed by a two colour flow cytometric assay. Annexin V and PI emissions were detected in the FL1 and FL2 channels of a FACS Calibur flow cytometer (Becton-Dickinson, USA), respectively. Flow cytometer data shows three distinct populations of cells. The normal healthy cells, early apoptosis, late apoptosis and necrotic populations were represented by annexin V-negative/PI-negative population, annexin V-positive/PI-negative, annexin V-positive/PI-positive and annexin-negative/PI-positive cells, respectively [11]. The data were analysed using Cell Quest program from Becton-Dickinson.

Assessment of Mitochondrial Membrane Potential

To measure the mitochondrial membrane potential (ΔΨm), 5,5',6,6'-tetrachloro-1,1',3,3'-tetraethylbenzimidazolylcarbocyanine iodide (JC-1), a sensitive fluorescent probe for ΔΨm was used [32]. The HeLa cells were treated with various concentration of NMK-TD-100 (0-10 μM) for 36 h. Cells were then rinsed with PBS twice, stained with 5 μM JC-1 for 30 min at 37°C. Cells were rinsed with PBS twice, resuspended in 1 mL PBS, and instantly assessed for red and green fluorescence with FACS Calibur flow cytometer (Becton-Dickinson, USA).

## Western Blot Analysis

Cultured HeLa cells (3 x 10^6^ cells /ml) were grown in the presence of NMK-TD-100 (0-10 µM) for 36 h. The respective cells were collected and extracted in cold lysis buffer (150 mM NaCl, 1% NP-40, 20 mM Tris-HCl, 20 μg/ml aprotinin, 20 μg/ml leupeptin, 1 mM orthovanadate, 2 mM PMSF, pH 7.4). The protein content of the extracts was estimated by Bradford method. Total protein (30-50 µg) form each sample was loaded in 10-12 % SDS-PAGE. The proteins were electrophoretically transferred on to polyvinylidene difluoride membrane, blotted with different monoclonal and polyclonal antibodies according to manufacturer’s mentioned dilution and subsequently with required secondary antibodies. Then, the membranes were exposed to Kodak X-ray films after chemiluminescent treatment.

Cell Cycle Analysis by Flow Cytometer

Cultured HeLa cells were grown at a density of 10^6^ cells /mL and incubated in the presence of NMK-TD-100 of varying concentrations (0-50 µM) for 24 h, respectively. After treatment, the cells were harvested, fixed in ice cold methanol for at least 30 min in 4°C, and incubated for 4 h at 37 °C in a PBS solution containing 1 mg/mL RNase A. Then nuclear DNA was labeled with propidium iodide (PI). Cell cycle analysis was performed using a Becton Dickinson FACS Caliber flow cytometer, and the data were analyzed using the Cell Quest program from Becton Dickinson [11].

Determination of the Mitotic Index

To evaluate mitotic indices [33], HeLa cells were grown up to a density of 3x10^4^cells/mL. Cells were then treated with NMK-TD-100 (0 – 5 µM), and incubated for 24 h. Cells were fixed with 10% formalin for 30 min, permeabilized in ice-cold methanol for 10 min, and stained with DAPI (1 µg/mL) to visualize nuclei. Mitotic indices were determined with the Olympus Fluorescence microscope. Results are the mean± SEM (standard error of the mean) of three experiments in each of which 1000 cells were counted at each concentration.

Sample Preparation for Confocal Microscopy

Cultured HeLa cells were grown at a density of 10^5^cells /ml, and treated with different concentration of NMK-TD-100 (0-5 µM) for 24 h. Subsequently, cells were washed twice by PBS, fixed by 2% para-formaldehyde, and incubated with permeable solution (0.1% Na-Citrate, 0.1% Triton) for 1 h. Nonspecific binding sites were blocked by incubating the cells with 5% BSA for 2 h at room temperature-controlled. Cells were then incubated with mouse monoclonal anti-α-tubulin antibody (1:200 dilutions) followed by anti mouse rhodamine conjugated IgG antibody (1:150 dilutions) and DAPI (1 μg/mL). After incubation, cells were washed with PBS, and viewed under a Ziess LSM 510 Meta confocal microscope [11].

Western Blot Analysis of Soluble and Insoluble Tubulin in Cellular System

The cellular tubulin polymerization was quantified by a modified method which was originally described by Minotti et al [34]. Cultured HeLa cells were treated with 0, 5 and 10 µM of NMK-TD-100 for 24 h. Then the cells were washed twice with PBS and harvested by trypsinization. Cells were lysed at 37°C for 5 min in the dark with 100 μL of hypotonic lysis buffer (1 mM MgCl_2_, 2 mM EGTA, 0.5% NP-40, 20 μg/mL aprotinin, 20 μg/mL leupeptin, 1 mM orthovanadate, 2 mM PMSF, and 20 mM Tris-HCl, pH6.8). After a brief but vigorous vortex, the samples were centrifuged at 14000 rpm (21000g) for 10 min. The 100 μL supernatants containing soluble (cytosolic) tubulin were separated from the pellets containing polymerized (cytoskeletal) tubulin. The pellets were resuspended in 100 μL of lysis buffer. The total concentrations of proteins in the soluble fraction and pellet fraction were estimated separately by the Bradford method. Equal amounts (50 μg) of each sample were added with SDS polyacrylamide gel electrophoresis sample buffer and run in a 10% SDS polyacrylamide gel. The sample was then analyzed by Western blotting and probed with the antibody against α-tubulin.

Tubulin Polymerization Assay

Tubulin (12 μM) was mixed with different concentrations of NMK-TD-100 (0-50 μM) in polymerization buffer (1 mM MgSO4, 1 mM EGTA, and 1.0 M monosodium glutamate, pH 6.8) and the polymerization reaction was initiated by incubating the tubulin-NMK-TD-100 complex at 37 °C just after addition of 1 mM GTP to the assembly. The rate and the extent of the polymerization reaction were monitored by light scattering at 350 nm [35] using a Jasco V-630 (Japan) spectrophotometer.

Depolymerization of preformed microtubules was monitored by light scattering at 350 nm in the presence of 0-50 µM NMK-TD-100. Tubulin (12 μM) was first polymerized in the assembly buffer for 20 min at 37°C, and different concentrations of NMK-TD-100 were then added to the reaction mixtures. The extent of the depolymerization reaction was monitored by light scattering at 350 nm using a Jasco V-630 spectrophotometer.

Electron Microscopy

Tubulin (10 µM) was polymerized in absence or presence of NMK-TD-100 (25 µM) at 37°C for 15 min as described above. The samples were fixed with pre-warmed 0.5% glutaraldehyde for 5 min, transferred to carbon coated grids and negatively stained with 1% uranyl acetate. Samples were visualized under Philips Fei Technai Spirit electron microscope

(1:1000 dilution).

Spectral Measurements

Absorbance measurements were performed in a JASCO (Tokyo, Japan) V-530 UV-visible spectrophotometer equipped with a Peltier temperature control system, using a cuvette of 1 cm path length. All fluorescence measurements were performed using a fluorescence spectrophotometer (Photo Technology Inc. USA, Model QM-4CW) equipped with a Peltier temperature control system. Fluorescence data was corrected for the inner filter effect according to equation of Lakowicz [36],

$F=F_{obs}antilog\text{ }\left[\left(A_{ex}+A_{em}\right)/2\right]$ …………………… (2)

where A_ex_ is the absorbance at the excitation wavelength and A_em_ is the absorbance at the emission wavelength.

Ligand-Tubulin Binding Measurements by Fluorescence Spectroscopy

The binding of the ligand to the protein was monitored by enhancement of ligand fluorescence in the presence of protein. Tubulin (0-15 µM) was incubated with fixed concentration of NMK-TD-100 (2 µM) for 30 min at 37°C along with a control set without any ligand. The excitation and emission wavelengths were 340 and 450 nm, respectively. The dissociation constant (K_d_) and stoichiometry of binding were calculated from the method described by Mas and Colman [37]. Tubulin (2 µM) was incubated with various concentration of NMK-TD-100 (0.1- 30 µM) for 30 mins at 37°C. The concentration of bound ligand was determined from the equation:

$\left[L\right]_{bound}=\left[L\right]_{total}\{\left(F/F_{0}-1\right)/\left(Q-1\right)\}$…………………………….. (3)

Where F_0_ and F were the fluorescence of the ligand in the absence and presence of tubulin respectively, [L]_bound_ was the concentration of bound ligand, [L]_total_ was the total concentration of ligand and Q was the fluorescence enhancement factor. Q was calculated by titrating fixed concentration of ligand (2 µM) with increasing concentration of tubulin (2.5- 15 µM). Q could be obtained from the intercept of the double reciprocal plot of (F/F_0_ -1) vs. [P], where [P] was the tubulin concentration. Free ligand concentration ([L]_free_ could be calculated from the difference between [L]_total_ and [L]_bound_. The dissociation constant K_d_ could be calculated from the Scatchard plot according to

$r/\left[L\right]_{free}=\left(r/K_{d}\right)-\left(n/K_{d}\right)$ ……………………………… (4)

where, r is the ratio of the concentration of bound ligand to the total protein concentration and

n represents the maximum number of binding sites.

Job Plot and Determination of Stoichiometry of Binding

The stoichiometry of binding was also determined using the method of continuous variation [38]. Several mixtures of tubulin and NMK-TD-100 were prepared by continuously varying concentrations of tubulin and NMK-TD-100 in the mixture, keeping the total concentration of NMK-TD-100 with tubulin constant at 5 μM. Reaction mixtures were incubated for 30 min at 37°C, and the fluorescence measurements at 450 nm were recorded using 340 nm as an excitation wavelength. A plot of F­_450_ vs. mole fraction of NMK-TD-100 was constructed to find out the stoichiometry of this binding reaction.

Binding Study Using Quenching of Tryptophan Fluorescence of Tubulin

Tubulin (1 µM) was incubated with varying concentrations of NMK-TD-100 (0- 50 µM) at 37 °C for 30 min. The fluorescence measurements were performed using 295 as the excitation wavelength to specifically excite the tryptophan residues of tubulin. When excited at 295nm, tubulin displayed a typical emission spectrum with a maximum at 335 nm, and NMK-TD-100 reduced the intrinsic fluorescence of tubulin. The apparent decrease in the fluorescence values in the presence of varying concentrations of NMK-TD-100 were corrected for the inner filter effect from equation [2]. The fraction of binding sites (X) occupied by NMK-TD-100 was determined using an equation X= (F_0_ - F)/F_max_, where F_0_ is the fluorescence intensity of tubulin in the absence of NMK-TD-100, *F* is the corrected fluorescence intensity of tubulin in the presence of NMK-TD-100, and F_max_ is calculated from the plot of 1/(F_0_  - F) versus 1/[NMK-TD-100] graph and extrapolating 1/[NMK-TD-100] to zero. The dissociation constant (K_d_) was determined using the relationship, (1/X) =1 + K_d_/L_f_, where L_f_ represents free NMK-TD-100 concentration, and L_f_ =*C* - *X*[Y], where *C* is total concentration of NMK-TD-100 and [Y] is the molar concentration of ligand-binding sites, assuming a single binding site per tubulin dimer [39].

Association Kinetics

The kinetics of the association of NMK-TD-100 with tubulin was measured under pseudo-first-order conditions, where the ligand was present in a large excess. Concentrations of NMK-TD-100 were 10, 20, and 30 μM, whereas the tubulin concentration was 1 μM. The ligand was added to the tubulin solution, and emission at 335 nm was measured upon excitation at 295 nm. The value ln (Qmax – Qt) was plotted against time, where Qmax is the maximum amount of quenched fluorescence, and Qt is the quenched fluorescence at time t. The biphasic plot was analyzed according to the method of Lambeir and Engelborghs [40],

$Qmax-Qt=Ae^{-\alpha t}+B$ ……………………………………. (5)

where A and B are amplitudes and α and β are observed rate constants of the fast and slow phases, respectively. The amplitude of the slow phase (B) was low relative to that of the fast phase (A). The apparent association rate constants (k_on_) were calculated as k_on_= α /c, where α is the slope of the plot of ln (Qmax – Qt) versus time t and c is the concentration of ligand.

Binding of NMK-TD-100 to the vinblastine Binding site of Tubulin

The ability of NMK-TD-100 to bind at the vinblastine binding site of tubulin was monitored by checking the change in NMK-TD-100- tubulin complex fluorescence [39]. Tubulin (2 µM) was incubated with different concentrations (0–50 µM) of vinblastine at room temperature for 20 min. Then 5 µM of NMK-TD-100 was added to all reaction mixtures and spectra were recorded after 30 min incubation at 37°C by exciting the samples at 340 nm.

Binding of NMK-TD-100 to Preformed tubulin–Colchicine Complex

Tubulin (2 µM) was incubated with varying concentrations of colchicine (0, 5, 10, 25, 50 and 100 µM) for 1 h at 37°C. Then, 5 µM NMK-TD-100 was added to the reaction mixtures and incubated for an additional 30 min. The dissociation constants for colchicine interaction with tubulin are reported to be 0.5 µM [39]. Therefore, under the experimental conditions used, tubulin would be completely liganded with colchicine. The reaction mixtures were excited at 390 nm and the emission spectra of enhancement of NMK-TD-100 fluorescence upon tubulin binding were recorded.

Molecular Modeling of NMK-TD-100-Tubulin Binding

The crystal structure of αβ-tubulin (PDB ID: 1JFF) was utilized as template for docking studies [41]. The molecular structure of NMK-TD-100 was drawn by using *chem draw* software and converted to mol file. The docking was performed using algorithm-based ligand docking program Discovery Studio 2.5. The energy minimized structures of tubulin (1JFF.pdb) and the ligand NMK-TD-100 were generated by using *prepare protein* module (using CHARMm force field) and *prepare ligand* module of Discovery Studio 2.5 respectively. In both cases all parameter were set to default values (Table S1). The lowest energy configuration of NMK-TD-100 was then docked into 42 different binding sites of the prepared protein molecule using standard parameters of Discovery Studio 2.5 throughout the simulation. The preferred site was determined calculating binding energy values. To predict whether colchicine interferes with the binding of NMK-TD-100, the crystal structure of αβ -tubulin (PDB ID: 1SA0) was utilized as template for this docking study [42]. 1SA0.pdb file represents the pdb file of colchicine bound tetrameric tubulin (stabilized by stathmin receptor). All the ligands associated with the above mentioned pdb file were removed from it and the protein was converted into a heterodimeric form prior to perform the simulation. Finally the energy minimized heterodimer was prepared by using prepare protein module (using CHARMm force field) of Discovery Studio 2.5. NMK-TD-100 (already prepared by using *prepare ligand* module) was then docked to its binding site (previously determined by using 1JFF.pdb file) on tubulin surface and the binding energy value in that site was calculated.

Statistical Analysis

Data are presented as the mean of at least three independent experiments along with Standard deviation (SD). Statistical analysis of data was done by one-way analysis of variance (ANOVA) with a Student’s t test, by using MS Excel, and two measurements were statistically significant if the corresponding p value was <0.05.

Results

Inhibition of Cell Proliferation by 5-(3-indolyl)-2-Substituted-1,3,4-Thiadiazoles

The anti-proliferative effect of some synthetic 5-(3-indolyl)-2-substituted-1,3,4-thiadiazoles was screened using human cervical epithelial carcinoma (HeLa) by MTT assay (data not shown) and it was found that NMK-TD-100 (Fig. 1A for Structure) was the most effective one among all the compounds in inhibiting the proliferation of the HeLa cells. NMK-TD-100 inhibited proliferation of HeLa cells in a concentration-dependent manner as measured (Fig. 2A). The IC_50_ value of NMK-TD-100 in HeLa cells was 1.42±0.11 µM for 48 h of treatment and the maximum proliferation inhibition of ≈ 90% occurred at the concentration of 100 µM. NMK-TD-100, being the most potent one in killing cancer cells was chosen for further work.

NMK-TD-100 was Less Toxic to Peripheral Blood Mononuclear Cells (PBMC)

The cytotoxicity of NMK-TD-100 in normal PBMC was examined and we found that it was less toxic towards PBMC compared to HeLa cells. The IC_50_ value of NMK-TD-100 in PBMC from healthy volunteers was 50±2.66 µM for 48 h, which was approximately 30-fold higher than the IC_50_ value of NMK-TD-100 in HeLa cells (Fig. 2B).

NMK-TD-100 Reduced the Colony Formation Ability of HeLa Cells

Cultured HeLa cells were treated with various concentrations of NMK-TD-100 (0, 2.5, 5 and 10 µM) for 48 h. After removal of the media, the cells were maintained for 10 days with triweekly change in media. After 10 days, the cells were fixed with 4% glutaraldehyde and stained with crystal violet. Each plate was photographed after removing the excess stain (Fig. 2C). Colony formation was quantitated by dissolving stained cells in Sorenson’s buffer (0.1 mol/L sodium citrate, 50% ethanol, pH 4.2) for colorimetric reading of OD at 550 nm. The spectrophotometric data showed that the % colony formation in HeLa cells treated with 2.5, 5 and 10 µM NMK-TD-100 were decreased to 43.55±6.15, 36.71±2.91 and 26.25±3.48%, respectively, with respect to that of control (Fig. 2D).

NMK-TD-100 Induced Apoptosis in HeLa Cells

To assess whether NMK-TD-100 induced apoptosis, treated cells were labeled with Annexin V-FITC/PI and analyzed by flow cytometer (Fig. 3A). The Annexin V positive/PI negative cells and Annexin V positive/PI positive cells were considered as early and late apoptotic cells, respectively. The numbers of early and late apoptotic cells were 7.55±3.29 and 5.62±1.54%, 13.80±4.35 and 5.82±2.67%, 22.52±5.12 and 7.92±1.83%, in case of HeLa cells treated with 2.5, 5, 10 µM of NMK-TD-100, respectively (Fig. 3A). In untreated control sample, the numbers of early and late apoptotic cells were only 1.80±0.78 and 0.99±0.37%, respectively.

.

NMK-TD-100 Induced Disruption of Mitochondrial Membrane Potential (ΔΨm) in HeLa Cells.

Chemically promoted apoptosis mediated by the mitochondria activation pathway is often associated with the collapse of ΔΨm as a result of depolarization and leakiness of the inner mitochondrial membrane. Therefore, to investigate whether mitochondrial membrane integrity is damaged by NMK-TD-100, change in mitochondrial membrane potential (ΔΨm) was measured using JC-1, a sensitive fluorescent probe for ΔΨm. When ΔΨm is low, JC-1 exists mainly in a monomeric form, which emits green fluorescence whereas it exists in aggregates and emits red fluorescence in high ΔΨm. As shown in Fig. 3B, NMK-TD-100 induced a collapse of mitochondrial membrane potential in a concentration dependent manner illustrated by the decrease in the ratio of red: green fluorescence. Thus the collapse of mitochondrial membrane potential may be an early event of NMK-TD-100 induced cell death.

NMK-TD-100 Caused the Change in Expression of Pro-apoptotic and Anti-apoptotic Proteins in HeLa cells.

The effect of NMK-TD-100 on the mitochondrial membrane potential intrigued us to study the major protein component of the mitochondrial apoptotic pathway. As shown in Fig. 3C NMK-TD-100 treatment, indeed, resulted in the increase in p53 level in HeLa cells accompanied by an increase in Bax(proapoptotic)/Bcl-2(antiapoptoic) ratio. Furthermore, we found that NMK-TD-100 treatment led to decrease in the amount of procaspase-3.

Effect of NMK-TD-100 on Cell Cycle Progression in HeLa Cells

The gradual accumulation of rounded cells was observed after treatment with NMK-TD-100, which resembled the morphology of mitotic cells. Thus the cell cycle progression status of NMK-TD-treated HeLa cells was analyzed. NMK-TD-100 induced G_2_/M arrest in HeLa cells in a dose-dependent manner (Fig. 4A). Nearly 49.91±5.67% of the cells were arrested at the G_2_/M phase of the cell cycle after treatment with 10 µM of NMK-TD-100 for 24 h, whereas the number of cells arrested in untreated control was 18.32±2.23%. When the cells were treated with 2.5 and 5 µM NMK-TD-100, 33.51±6.11 and 42.66±4.63 % population of cells were arrested at G_2_/M phase, respectively.

Determination of Mitotic index in NMK-TD-100 Treated HeLa Cells

In the aim of confirming the deleterious effect of NMK-TD-100 in cell cycle progression in HeLa cells, the mitotic indices were determined in HeLa cells in the absence and presence of NMK-TD-100. Confocal microscopic analysis of fixed and DAPI stained HeLa cells revealed that 5.75±1.91% of the cells were in mitosis in the absence of NMK-TD-100, whereas in presence of 2.5 and 5 µM NMK-TD-100, 21.98±2.59 and 38.55±4.81% of HeLa cells were halted at the mitotic phase of the cell cycle, respectively (Fig. 4B-4E). The distribution of the HeLa cells in different mitotic phases (Table 1) showed that cells were mainly blocked on the prophase/pro-metaphase and metaphase upon NMK-TD-100 treatment, whereas the accumulation at anaphase and telophase was comparatively low. Together, all data indicated that NMK-TD-100 arrested HeLa cells in mitosis.

Effect of NMK-TD-100 on Cellular Microtubule Structure

As NMK-TD-100 caused cell cycle arrest at G_2_/M phase, we were interested to investigate whether there was any change in microtubule cytoskeletal structure of HeLa cells in the presence of NMK-TD-100. Concentration dependent addition of NMK-TD-100 (0-5 µM) to HeLa cells caused a substantial reduction in the number of interphase microtubules at the periphery and disorganization of central networks of the cells was observed (Fig. 5A-5I). The spindle microtubules were equally disrupted in HeLa cells in the presence of varying concentration of NMK-TD-100 (Fig. 5J-5O). Control cells in metaphase displayed a normal bipolar mitotic spindle (Fig. 5J). Cells treated with NMK-TD-100 for 24 h displayed disrupted appearance of mitotic spindles (Fig 5L, 5N). Formation of multipolar mitotic spindle was also observed in HeLa cells after NMK-TD-100 exposure. Thus, NMK-TD-100 effectively disrupted the interphase and spindle microtubule in HeLa cells.

NMK-TD-100 Inhibited the Reassembly of Cold Depolymerized Microtubule in HeLa Cells

Cellular microtubules (Fig. 6A) were completely depolymerized by incubating the cells at 4°C for 3 h (until the cells appeared spherical as shown in Fig. 6B). The cold media were replaced by warm media in the absence or presence of NMK-TD-100 and kinetics of reformation of interphase microtubules were studied. In normal media the microtubule reassembled within 3 h of incubation with warm media and the cells appeared almost normal in shape (Fig. 6C) with respect to the microtubule network in control cells (Fig. 6A). However, in the presence of 2.5 and 5 µM of NMK-TD-100 (Fig.6D, 6E), the interphase microtubules failed to reform even after 3 h of incubation in warm media. The results showed that NMK-TD-100 inhibited the reassembly of microtubules in HeLa cells.

NMK-TD-100 Reduces the Insoluble Fraction of Microtubule Mass in HeLa Cells

In order to show the effect of NMK-TD-100 on soluble and insoluble fraction of tubulin in HeLa cells, western blot was performed in order to visualize the change in soluble tubulin fraction and polymerized microtubule mass with respect to total tubulin in HeLa cells after treatment with NMK-TD-100. After 24 h treatment with 0- 10 µM NMK-TD-100 (Fig. 6F), there was a gradual decrease in insoluble polymerized microtubule mass with concomitant increase in soluble fraction in HeLa cells. However, there was no change in the total tubulin mass as detected by using anti-α-tubulin antibody. These results indicated that NMK-TD-100 depolymerized microtubule mass into tubulin dimers in HeLa cells.

Inhibition of Tissue Purified Tubulin Polymerization by NMK-TD-100

Since we observed that NMK-TD-100 affects the cellular architecture of HeLa cells by perturbing the intracellular microtubular network, we examined the effect of NMK-TD-100 on tubulin polymerization using purified tubulin and determined the polymerization inhibitory potential of the ligand. We investigated the concentration-dependent effect of NMK-TD-100 on polymerization ability of purified tubulin into microtubule. Purified tubulin (12 µM) was polymerized in the presence of different concentrations of NMK-TD-100 (0- 50 µM) as described in the Experimental Procedures. NMK-TD-100 inhibited the rate and extent of tubulin polymerization in a concentration-dependent manner (Fig. 7A). For example, the level of inhibition of polymerization was 69%, when 30 μM NMK-TD-100 was added. The percentage of inhibition of microtubule polymerization was calculated using the steady-state absorbance readings in the absence and presence of different concentrations of NMK-TD-100 and the inhibitory concentration of microtubule polymerization (IC_50_) occurred at NMK-TD-100 concentration of 17.5±0.35 μM.

The inhibition of tubulin polymerization in the presence of NMK-TD-100, as monitored by light scattering assay, could be due to the formation of very short microtubules, altered polymer morphology, or the formation of small aggregates of tubulin. To differentiate among these possibilities, reaction products formed in the absence and presence of NMK-TD-100 were examined with a transmission electron microscope (Fig. 7B). In the absence of NMK-TD-100 (control experiment), tubulin polymerized in 1 M glutamate buffer formed typical microtubule polymers, which is consistent with previous reports. In the presence of 25 µM NMK-TD-100, fewer and shorter microtubules were observed compared to the control set, indicating robust inhibition of microtubule formation. Moreover, there was formation of visible aggregates of tubulin in presence of NMK-TD-100.

We also investigated whether NMK-TD-100 could induce depolymerization of preformed microtubules in a *cell-free system*. We monitored decrease of absorbance at 350 nm as a measure of depolymerization of preformed microtubules. Tubulin (1.2 mg/ml) was polymerized in the presence of 1 M glutamate, 1mM GTP for 20 min at 37 °C, different concentrations of NMK-TD-100 were added to the preformed microtubule suspensions and the change in optical density was studied. NMK-TD-100 induced rapid depolymerization of preformed microtubules in a concentration-dependent fashion, as indicated by the decrease in the absorbance values at 350 nm (Fig. 7C).

Promotion of NMK-TD-100 Fluorescence upon Binding to Tubulin

Binding of NMK-TD-100 (2 μM) to tubulin enhanced its fluorescence intensity and showed a blue shift in the emission maxima with an increase in the concentration of protein(0-15 μM) (Fig. 8A). Enhancement of fluorescence on binding of a small ligand to a protein is generally explained as arising due to the hydrophobic environment provided by the binding site, thus lowering the probability of mobile dipole-dependent internal conversion processes [43]. The change in polarity of solvent can also facilitate such fluorescence enhancement of the ligand. This fact was supported by the observation that the fluorescence of NMK-TD-100 (20 μM) increased significantly along with a large blue shift, when the solvent was changed from water to methanol (Fig. 8B). The stoichiometry and the dissociation constant (K_d_) of the tubulin-NMK-TD-100 interaction had been estimated by measuring NMK-TD-100 fluorescence enhancement. From the titration curve (Fig. 8C) of a constant amount of tubulin (2 μM) and various concentrations of ligand (0.1-30 μM), the binding data were analyzed by Scatchard plot as described in Experimental Procedures, and the analysis of the data yielded a linear plot with a dissociation constant of 1.26±0.32 µM and a stoichiometry of 1.07±0.15. The stoichiometry of the drug-protein complex was determined using the Job plot. In this plot, concentrations of both tubulin and NMK-TD-100 were varied while the total drug-protein concentration was kept fixed at 5 μM. Results of such experiments are shown in Fig. 8D. The stoichiometry of binding calculated by using this method of continuous variation was found to be 1:1.This stoichiometry of protein:ligand binding obtained from Scatchard plot matched very well with the data obtained from Jobs Plot. Taken together, these data suggested that NMK-TD-100 bound to tubulin at a single site.

Binding of NMK-TD-100 to tubulin- quenching of intrinsic tryptophan residues of tubulin

The binding of NMK-TD-100 to tubulin was studied by measuring the quenching of the intrinsic tryptophan fluorescence of tubulin. NMK-TD-100 quenched the tryptophan fluorescence of tubulin in a time- and concentration -dependent manners (Fig. 9A). Addition of 10 μM NMK-TD-100 to tubulin (1 μM) quenched almost 35% of the tryptophan fluorescence of tubulin during 30 min incubation at 37ºC. Again 30 µM NMK-TD-100 quenched nearly 45% of trytophan fluorescence of tubulin (1µM) upon incubation of 30 mins at 37°C. The dissociation constant (K_d_) of the tubulin-NMK-TD-100 interaction was estimated by measuring the effects of NMK-TD-100 on the intrinsic tryptophan fluorescence of tubulin. The double-reciprocal plot of the binding data (Fig. 9B, 9C) yielded a dissociation constant of 1.01±0.42 µM, which was almost similar to the K_d_ value obtained by the ligand fluorescence titration (Fig 8C).

The kinetics of the NMK-TD-100-tubulin association reaction was studied monitoring the quenching of intrinsic protein fluorescence under pseudo-first-order condition, with NMK-TD-100 used at least 10-fold molar excess. Fig. 9D shows the kinetic profile for the binding of NMK-TD-100 to tubulin under pseudo first-order conditions for 20 min at 37°C. The quenching data were analyzed using a biexponential equation as explained in experimental procedures. The apparent association rate constant values calculated from the equation as mentioned in the Experimental procedure were 1747.02±97.51 M^-1^s^-1^ for the major fast phase and 53.66±17.95 M^-1^s^-1^ for the minor slow phase at 37°C, taking the average of nine consecutive experimental data analyses at three different concentrations (10, 20, and 30 μM) of NMK-TD-100. Under the same experimental condition, the apparent association rate constant (k_on_) calculated for association of colchicine with tubulin using the biexponential equation was 155.9±23.32 M^-1^s^-1^ at 37°C.

Characterization of Binding Site for NMK-TD-100

We used the NMK-TD-100–tubulin complex fluorescence to determine whether NMK-TD-100 could bind to the colchicine or vinblastine site on tubulin. Tubulin (2 µM) was incubated with varying concentration of colchicine for 1 h at 37°C. NMK-TD-100 was added to the tubulin-colchicine complex and fluorescence of NMK-TD-100 in different samples was monitored at 450 nm after exciting at 390 nm. We checked the fluorescence values of tubulin and colchicine complexes at 450 nm after excitation at 390 nm and found them to be very low as compared to tubulin and NMK-TD-100 complex, and those values were subtracted from those of the corresponding fluorescence values of tubulin-NMK-TD-100- colchicine complex. We hypothesized that if the ligand could inhibit the binding of NMK-TD-100 to tubulin, it should cause decline in the development of NMK-TD-100–tubulin complex fluorescence. The extent of NMK-TD-100 binding at various concentrations of colchicine is shown in Fig. 10B and 10C and the time dependent study using colchicine (40 µM) is shown in Fig. 10A. Colchicine inhibited the fluorescence of tubulin–NMK-TD-100 complex to certain extent shown from both time and concentration dependent study. For example, 50µM colchicine inhibited NMK-TD-100 binding to tubulin by ~20%. These data indicated that the binding site of NMK-TD-100 on tubulin may partly overlap with the binding site of colchicine. Alternatively, the binding of colchicine to tubulin induced conformational changes in tubulin that reduced NMK-TD-100 binding to the protein. Vinblastine did not affect the tubulin–NMK-TD-100 complex fluorescence indicating that NMK-TD-100 bound to tubulin at a site which was different from that of vinblastine (Fig. 10D).

*In-silico* Prediction of NMK-TD-100 Binding to Tubulin

To gain further insights into the binding site of NMK-TD-100 in tubulin, computation based docking analysis was performed with tubulin–NMK-TD-100 complex. For determination of the binding site of NMK-TD-100 on tubulin surface, the ligand was docked on tubulin surface by using discovery studio 2.5. Based on the highest negative value of binding energy (-97.39 Kcal/mol), the most stable tubulin-NMK-TD-100 complex was found to be formed when the ligand was docked on site 1 (according to the software), located between α and β subunit of tubulin (Fig. 11A, Table S1 for binding parameters). Further analysis revealed that this preferred site is located just adjacent to the colchicine binding site of tubulin (Fig. 11 D). NMK-TD-100 binding was stabilized by several polar, nonpolar, and charged residues inside the pocket which is made up of beta sheets (particularly βI, βII, βIII, βIV, βV, βVI and its associated loops) of α subunit and T7 loop and alpha helix (H8) of β subunit (Table 2) which constitutes the binding pocket (Fig. 11B). Additionally, the tubulin-NMK-TD-100 binding was strengthened by hydrogen bonding with Lys254 of β subunit and Asn228 of α subunit (Fig. 11C). The binding of NMK-TD-100 to the colchicine binding site of the tubulin was also performed using 1JFF.pdb file in order to determine its affinity to the above mentioned site. The binding energy at colchicine binding site of tubulin was found to be -46.15 K cal/mol and the value showed lower affinity of NMK-TD-100 to colchicine binding site of tubulin as compared to the site 1.

For determining the effect of colchicine on tubulin-NMK-TD-100 binding, NMK-TD-100 was docked on its binding site (previously determined by using 1JFF.pdb file) in 1SA0.pdb file (which is colchicine bound tubulin). Subsequently binding energy at the preferred site was calculated using Discovery Studio 2.5. and it was found the binding energy (-71.82 kcal/mol) is less negative compared with when docked to site 1 of pdb 1JFF. This indicates that binding of colchicine can partly interfere with binding of NMK-TD-100 to tubulin.

It was also previously reported that colchicine binding on tubulin surface was stabilized by amino acids present in H7, H8 and T7 loops of β subunits of tubulin. The binding pocket of NMK-TD-100 also constitutes of the H8 and T7 loop of the β subunit of tubulin. Furthermore Lys 254 of β subunit which was found to be hydrogen bonded with NMK-TD-100, also interacted with colchicine by non covalent interactions other than hydrogen bond. Moreover, during NMK-TD-100 and tubulin interaction, Lys 254 also formed hydrogen bond with Asn 258 and non-covalently interacted with other amino acid residues like Cys 241, Leu 242, Ala 250. These amino acid residues were also important for stabilization of colchicine into its binding sites. So it can be inferred that NMK-TD-100 binds to tubulin very near to the colchicine binding site and also colchicine interferes with NMK-TD-100 binding to tubulin.

Discussion

In this work, a group of 5-(3-indolyl)-2-substituted-1,3,4-thiadiazoles [14] was screened for their cytotoxicity in HeLa cells. Among the 5-(3-indolyl)-2-substituted-1,3,4-thiadiazole, NMK-TD-100 was found to be the most potent one in inhibiting the proliferation of HeLa cells. However, it should be noted that its toxicity in normal PBMC from healthy volunteer was significantly lower than that in HeLa cells. NMK-TD-10 induced apoptosis in HeLa cells through intrinsic mitochondrial pathway. We wanted to know the target for NMK-TD-100 in HeLa cells, whose alteration in structure and function caused apoptosis.

Results of cell cycle progression experiment in the presence of NMK-TD-100 demonstrated that it induced arrest of cell cycle progression at G_2_/M phase effectively after 24 h of treatment (Fig. 4A) and that prompted us to investigate whether NMK-TD-100 targeted microtubule in cells. Indeed, we observed that NMK-TD-100 depolymerized microtubule network in HeLa cells (Fig. 5) and that was early event of NMK-TD-100-induced apoptosis. We monitored kinetics of the events in HeLa cells after treatment of the ligand. When cells were treated with NMK-TD-100 for 24 h, no significant induction of apoptosis and change in MMP were observed (data not shown). However, a significant amount of depolymerization of microtubule structure was observed (Fig. 5) and as a consequence cells were arrested at the G_2_/M phase of the cell cycle in 24 h ligand-treated cells (Fig. 4A). Moreover, accumulation of HeLa cells at G_2_/M phase of cell cycle with concomitant increase in number of mitotic cells (Fig. 4B-4E) and formation of deformed and multipolar spindles were observed (Fig. 5J-5L). Again continuous arrest of cell cycle at G_2_/M phase led to induction of apoptosis via intrinsic pathway that was evident when we analyzed the results of 36 h NMK-TD-100-treated HeLa cells (Fig. 3A). Significant amount of apoptosis and decline in MMP observed (Fig. 3B), increase in p53 protein level, increase in ratio of bax/bcl-2 and activation of caspase-3 occurred in 36 h treated cells observed (Fig. 3C). Incubation of NMK-TD-100 in HeLa cells for 48 h reflected that NMK-TD-100 treatment reduced colony formation ability in HeLa cells (Fig. 2C-2D) .

One of the important properties of microtubule is that it depolymerizes and repolymerizes in cells and also in a *cell-free system* in a temperature dependent manner. Agents, which depolymerize microtubules in cells, inhibit this reassembly process [11]. When HeLa cells were incubated at 4°C for 3 h, the microtubule network was completely depolymerized (Fig. 6B). When these cells were incubated in warm media at 37°C in the absence of NMK-TD-100, depolymerized microtubules again repolymerized to form regular structure (Fig. 6C). However, when these cells were incubated in warm media containing NMK-TD-100, reassembly of microtubules was significantly deterred (Fig. 6D-6E) and amount of soluble tubulin was increased (Fig. 6F). These results also clearly indicated that NMK-TD-100 targeted microtubules in HeLa cells.

NMK-TD-100 not only depolymerized microtubules and inhibited reassembly of depolymerized microtubules in HeLa cells but also inhibited tissue purified tubulin assembly in a *cell-free system* (Fig. 7A). The IC_50_ value for inhibition of purified tubulin polymerization was 17.5±0.35 μM, whereas cell viability results indicated that the IC_50_ value was 1.42±0.11 µM for NMK-TD-100. These large difference in IC_50_ values are also observed in other microtubule targeting agents e.g., colchicine, a very well-known anti-microtubule agent, which also shows a large difference in IC_50_ values for cell viability in cancer cells (around 50-60 nM) and for inhibition of purified tubulin polymerization (around 1 μM). The probable reason is that solution environments are different in cellular system and in a *cell-free system*.

Fluorescence spectroscopy is a very powerful tool for studying protein-ligand interaction and this technique has been extensively used for tubulin-ligand binding study [11,39,44]. Any ligand which binds to purified tubulin may cause enhancement of its fluorescence or quenching of intrinsic tubulin fluorescence as tubulin contains 12 tryptophan residues which are heterogeneously oriented in the α and β tubulin heterodimer. There was a marked enhancement in fluorescence of NMK-TD-100 in the presence of tubulin along with a noticeable blue shift in ligand emission maxima (Fig. 8A). This phenomenon can be interpreted as protein binding induced enhancement of fluorescence of small ligand attributed due the hydrophobic nature of the binding site as this increase in emission is also observed with change in polarity of solvent. The fluorescence enhancement of NMK-TD-100 was observed when the solvent was changed from water to methanol accompanied by large blue shift in emission maxima (Fig. 8B). It is known that shift in emission maxima may arise due the molecular motion i.e. the solvent molecules get reoriented at the excited state dipole and prodan can be cited as the classic example of such phenomenon [43]. Whether such phenomenon is also operating behind the above mentioned characteristic of NMK-TD-100 needs further investigation. Binding study using enhancement of NMK-TD-100 fluorescence upon binding to tubulin revealed that it bound to tubulin at a single site with a K_d_ value of 1.26±0.32 µM (Fig. 8C-8D). Similar binding affinity (1.014±0.42 µM) was also obtained when results of quenching of tryptophan fluorescence of tubulin upon binding of NMK-TD-100 were analyzed (Fig. 9A-9C). These results indicated that NMK-TD-100 had strong and robust affinity towards tubulin.

The kinetics of the NMK-TD-100-tubulin association reaction was studied by monitoring quenching of tryptophan fluorescence under pseudo-first-order conditions, and the best fit was obtained with a biexponential function (Fig. 9D). The NMK-TD-100-tubulin binding follows the biphasic kinetic same as that of colchicine-tubulin binding. However, the value of apparent second order rate constant for NMK-TD-100 binding tubulin is ~10-fold faster than that of colchicine binding to tubulin. It is already reported that the characteristic biphasic binding of colchicine to tubulin can be attributed to the presence of different kinds of isotypes in unfractionated tubulin and the differential binding of colchicine to them [45]. Many of the tubulin targeted anticancer agents which inhibit tubulin polymerization, bind to the colchicine or vinblastine binding site on the tubulin or near its vicinity. As binding rate of NMK-TD-100 with tubulin was 10-fold faster than colchicine-tubulin reaction, and colchicine binds to tubulin irreversibly [44,45] , we checked NMK-TD-100 binding with preformed tubulin-colchicine complex. NMK-TD-100 binding to tubulin was partially inhibited by prior incubation of tubulin with colchicine (Fig. 10A-10C). However, vinblastine had no effect on NMK-TD-100-tubulin binding (Fig. 10D). This data was validated by the *in silico* modeling study of NMK-TD-100- tubulin interaction (Fig. 11). From the docking studies, it can be inferred that NMK-TD-100 binds to a site which is partly overlapping with the A-ring of colchicine binding site of tubulin as NMK-TD-100 has identical A-ring like structure as that of colchicine (Fig. 1A). NMK-TD-100 affinity to the preferred site diminished in tubulin molecule which was initially bound to colchicine. This molecular modeling data corroborates with the fluorescence spectroscopic study of NMK-TD-100 binding to tubulin with prior incubation with colchicine.

In conclusion, we have identified a novel microtubule-targeted anticancer agent NMK-TD-100. Cellular data explores its mitotis blocking activity in cancer cells which may be the early event to its anti-proliferative activity and apoptosis induction. The interaction of NMK-TD-100 with purified tubulin is also well studied. All the above studies indicate that NMK-TD-100 may have a chemotherapeutic potential and further study is required for unraveling its efficacy as anti- cancer agent.

Acknowledgement

Confocal Microscope and FACS instrument facilities are developed by grants from National Common Minimum Project, Govt. of India.

References:

1. Chhabra S, Bhavani M, Mahajan N, Bawaskar R (2010) Cervical cancer in Indian rural women: trends over two decades. J Obstet Gynaecol 30: 725-728.
2. **Canavan TP, Doshi NR (2000). "Cervical cancer". Am Fam Physician 61: 1369–1376.**
3. **"FDA Approves First Drug Treatment for Late-Stage Cervical Cancer". U.S. Food and Drug Administration. 2006-06-15.**
4. Calligaris D, Verdier-Pinard P, Devred F, Villard C, Braguer D, et al. (2010) Microtubule targeting agents: from biophysics to proteomics. Cell Mol Life Sci 67: 1089-1094.
5. Nogales E, Wang HW (2006) Structural intermediates in microtubule assembly and disassembly: how and why? Curr Opin Cell Biol 18: 179–184.
6. Kline-Smith SL, Walczak CE (2004) Mitotic spindle assembly and chromosome segregation: refocusing on microtubule dynamics. Mol Cell 15: 317–327.
7. Jordan MA, Wilson L (2004) Microtubule as a target for anticancer drugs. Nat Rev Cancer 4: 253–265.
8. Tian W, Qin L, Song Q, He L, Ai M, et al. (2010) A Novel Synthetic Analog of 5, 8-Disubstituted Quinazolines Blocks Mitosis and Induces Apoptosis of Tumor Cells by Inhibiting Microtubule Polymerization. PLOS ONE 5: 10499.
9. Torfs S, Cadron I, Amant F, Leunen K, Berteloot P, **et al.** (2012) Evaluation of paclitaxel/carboplatin in a dose dense or weekly regimen in 66 patients with recurrent or primary metastatic cervical cancer. Eur J Cancer 48: 1332-1340.

# Morris M, Blessing JA, Monk BJ, McGehee R, Moore DH (2004) **Phase II study**of**cisplatin**and**vinorelbine**in**squamous cell carcinoma**of the**cervix**: a**gynecologic oncology group study**. J Clin Oncol 22: 340-344.

1. Acharya BR, Choudhury D, Das A, and Chakrabarti G (2009). Vitamin K3 disrupts the microtubule networks by binding to tubulin: a novel mechanism of its antiproliferative activity. Biochemistry 48: 6963-6974.
2. Fojo AT, Ueda K, Slamon DJ, Poplack DG, Gottesman MM, et al. (1987) Expression of a multidrug-resistance gene in tumors and tissues. Proc Natl Acad Sci USA 84: 265–269.
3. Buolamwini JK, Addo J, Kamath S, Patil S, Mason D, et al. (2005) Small molecule antagonists of the MDM2 oncoprotein as anticancer agents. Curr Cancer Drug Targets 5: 57-68.
4. Kumar D, Maruthi NK, Chang KH, Shah, K (2010) Synthesis and anticancer activity of 5-(3-indolyl)-1,3,4-thiadiazoles. Eur J Med Chem 45: 4664-4668
5. **Shen**L,**Zhang**Y,**Wang**A,**SieberMcMaster**E,**Chen**X,**et al.** (2007) Synthesis and identification of 1,2,4- thiadiazole derivatives as a new series of potent and orally active dual agonists of peroxisome proliferator-activated receptors alpha and delta. J Med Chem **50**: 3954-3963.

# Kadi AA, El-Brollosy NR, Al-Deeb OA, Habib EE, Ibrahim TM, et al. (2007) **Synthesis**,**antimicrobial**, and**anti-inflammatory activities**of**novel 2**-(**1-adamantyl**)-**5-substituted-1**,**3**,**4-oxadiazoles**and**2**-(**1-adamantylamino**)-**5-substituted-1**,**3**,**4-thiadiazoles**.

Eur J Med Chem 42: 235-342.

1. **Turner**S,**Myers**M,**Gadie**B,**Nelson**AJ,**Pape**R,**et al.** (1988) Antihypertensive thiadiazoles. 1. Synthesis of some 2-aryl-5-hydrazino-1,3,4-thiadiazoles with vasodilator activity. J Med Chem 31: 902-907.
2. Foroumadi A, Mansouri S, Kiani Z, Rahmani A (2003) Synthesis and in vitro antibacterial evaluation of N-[5-(5-nitro-2-thienyl)-1,3,4-thiadiazole-2-yl] piperazinyl quinolones. Eur J Med Chem 38: 851-854.
3. Dogan HN, Duran A, Rollas S, Sener G, Uysal MK, et al. (2002) Synthesis of new 2,5-disubstituted-1,3,4-thiadiazoles and preliminary evaluation of anticonvulsant and antimicrobial activities. Bioorg Med Chem 10: 2893-2898.
4. Yusuf M, Khan RA, Ahmed B. (2008) Syntheses and anti-depressant activity of 5-amino-1, 3, 4-thiadiazole-2-thiol imines and thiobenzyl derivatives. Bioorg Med Chem 16: 8029-8034.
5. Poorrajab F, Ardestani SK, Emami S, Behrouzi-Fardmoghadam M, Shafiee A, et al. (2009) Nitroimidazolyl-1,3,4-thiadiazole-based anti-leishmanial agents: synthesis and in vitro biological evaluation. Eur J Med Chem 44: 1758-1762.
6. Padmavathi V, Sudhakar Reddy G, Padmaja A, Kondaiah P, Ali-Shazia (2009) Synthesis, antimicrobial and cytotoxic activities of 1,3,4-oxadiazoles, 1,3,4-thiadiazoles and 1,2,4-triazoles. Eur J Med Chem 44:2106-2112.
7. Matysiak J (2007) Evaluation of electronic, lipophilic and membrane affinity effects on antiproliferative activity of 5-substituted-2-(2,4-dihydroxyphenyl)-1,3,4-thiadiazoles against various human cancer cells. Eur J Med Chem 42: 940-947.
8. Asbury R, Blessing JA, Moore D (1996) A phase II trial of aminothiadiazole in patients with mixed mesodermal tumors of the uterine corpus: a Gynecologic Oncology Group study. Am J Clin Oncol 19: 400-402.
9. Peifer C, Selig R, Kinkel K, Ott D, Totzke F, et al. (2008) Design, synthesis, and biological evaluation of novel 3-aryl-4-(1H-indole-3yl)-1,5-dihydro-2H-pyrrole-2-ones as vascular endothelial growth factor receptor (VEGF-R) inhibitors. J Med Chem 51: 3814-3824.
10. Garuti L, Roberti M, Pession A, Leoncini E, Hrelia S (2001) Synthesis and antiproliferative activity of some thiazolylbenzimidazole-4,7-diones. Bioorg Med Chem Lett 11: 3147-3149.
11. Hamel E, Lin CM (1981) Glutamate-induced polymerization of tubulin: Characteristics of the reaction and application to the large scale purification of tubulin. Arch Biochem Biophys 209: 29–40.
12. Bradford MM (1976) A rapid and sensitive method for the quantitation of microgram quantities of protein utilizing the principle of protein-dye binding. Anal Biochem 72: 248–254.
13. Dariusz S, Sarah JS, Richard HC, Michael B (1993) An improved MTT assay. J. Immunol .Methods 157: 203-207.
14. Paino IM, Marangoni VS, de Oliveira Rde C, Antunes LM, Zucolotto V (2012) **Cyto**and**genotoxicity**of**gold nanoparticles**in**human hepatocellular carcinoma**and**peripheral bloodmononuclear cells**. Toxicol Lett 215: 119-125.

# Xie Z, Choong PF, Poon LF, Zhou J, Khng J, et al. (2008) **Inhibition**of**CD44 expression**in**hepatocellular carcinoma cells enhances apoptosis**,

# **chemosensitivity**, and **reduces tumorigenesis**and**invasion**. Cancer Chemother Pharmacol 62: 949-957.

1. Li J, Qin Z, Liang Z (2009). The prosurvival role of autophagy in Resveratrol-induced cytotoxicity in human U251 glioma cells. BMC Cancer 9: 215-.
2. Kamath K, Jordon MA (2003) Suppression of microtubule dynamics by Epothilone B is associated with mitotic arrest. Cancer Res 63: 6026–6031.
3. Minnoti AM, Barlow BS, Cabral F (1991) Resistance to antimitotic drugs in chinese hamster ovary cells correlates with changes in the level of polymerized tubulin. J Cell Biochem 266: 3987–3994.
4. Gaskin F, Cantor CR, Shelanski ML (1974) Turbidimetric studies of the in vitro assembly and disassembly of porcine neurotubules. J Mol Biol 89: 737–755.
5. Lakowicz JR (1999) Principles of Fluorescence Spectroscopy, 2^nd^ ed., Kluwer Academic/Plenum Publishers, New York.
6. Mas MT, Colman RF (1985). Spectroscopic studies of the interactions of coenzymes and coenzyme fragments with pig heart, oxidized triphosphopyridine nucleotide specific isocitrate dehydrogenase. Biochemistry 24: 1634–1646.
7. Ward LD (1985) Measurement of ligand binding to protein by fluorescence spectroscopy. Methods Enzymol 117: 400–414.
8. Gupta KK, Bharne SS, Rathinasamy K, Naik NR, Panda D (2006) Dietary antioxidant curcumin inhibits microtubule assembly through tubulin binding. Febs J 273: 5320-5332.
9. Lambeir A, Engelborghs Y (1981) A fluorescence stopped flow study of colchicine binding to tubulin. J Biol Chem 256: 3279–3282.
10. Lowe J, Li H, Downing KH, Nogales E (2001) Refined structure of alpha beta-tubulin at 3.5 A resolution. J Mol Biol 313: 1045-1057
11. Ravelli RB, Gigant B, Curmi PA, Jourdain I, Lachkar S, el al. (2004) Insight into tubulin regulation from a complex with colchicine and a stathmin like  domain. Nature 428: 198-202.
12. Chattopadhyay A, Rawat SS, Kelkar DA, Ray S, Chakrabarti A (2003) Organization and dynamics of tryptophan residues in erythroid spectrin: Novel structural features of denatured spectrin revealed by the wavelength-selective fluorescence approach. Protein Sci 12: 2389–2403.
13. Bhattacharyya B, Kapoor S, Panda D (2010) F**luorescence spectroscopic methods**to**analyze drug-tubulin interactions.** Methods Cell Biol 95: 301-329.
14. Banerjee A, Luduena RF (1992) Kinetics of colchicine binding to purified β tubulin isotypes from bovine brain. J Biol Chem 267: 13335–13339.

Figure Legend

Figure 1. NMK-TD-100- Structure and absorption spectrum. (A) Structure of NMK-TD-100 and colchicine (B) The absorption spectrum of NMK-TD-100 (100 µM).

Figure 2. Cytotoxicity of NMK-TD-100 in HeLa and PBMC cells. (A) HeLa cells were cultured for 48 h with various concentrations (0-100 µM) of NMK-TD-100. Cell viability was assessed by MTT assay and is expressed as a percentage of control. Data are represented as the mean±SD. [*p<0.05 vs. control, where n=4]. (B) Percentage of cell death in freshly isolated PBMC after treatment with NMK-TD-100 for 48 h. Data are represented as the mean±SD [*p<0.05 vs. control, where n=3]. (C, D) Colony formation assay. Cultured HeLa cells were seeded in six-well plates at a density of 1,000 cells per well and cells were treated with NMK-TD-100 (0-10 µM) for 48 h. NMK-TD-100 containing media was replaced with fresh media and subsequently cells were cultured for 10 days. At the end, cells were fixed and stained with crystal violet, images were taken. Colony formation was quantified by dissolving stained cells in Sorenson’s buffer for colorimetric reading of OD at 550 nm. Data are represented as the mean±SD [*p<0.05 vs. control, where n=3].

Figure 3. NMK-TD-100 induced mitochondrial pathway mediated apoptosis in HeLa cells. (A) Annexin V-FITC /PI assay for showing that NMK-TD-100 induced apoptosis in HeLa cells. Cultured HeLa cells were treated with 0- 10 µM NMK-TD-100 for 36 h, cells were harvested and stained with annexin V-FITC and PI. The percentage of early apoptotic cells in the lower right quadrant (annexin V-FITC positive/PI negative cells), as well as late apoptotic cells located in the upper right quadrant (annexin V-FITC positive/PI positive cells). (B) NMK-TD-100 induced collapse of mitochondrial membrane potential in HeLa cells. Cells were treated with 0-10 µM NMK-TD-100 for 36 h. Then treated-cells were stained with JC1 and analyzed using flow cytometer. Red fluorescence emitted from the cells with normal mitochondria (red population in figure) gradually decreases with concomitant increase in green fluorescence emitted from that containing declined mitochondrial membrane potential (green population in figure). (C) Western Blot analysis of change in expression of pro and anti-apoptotic proteins (p53, bax, bcl2 and procaspase 3) of 36 h ligand-treated HeLa cells. Probing of α- tubulin was used as a loading control. The results represent the best of data collected from three experiments with similar results.

Figure 4. Cell cycle progression analysis and determination of mitotic index in NMK-TD-100 treated HeLa cells. (A) Effect of NMK-TD-100 on cell cycle progression of HeLa cells. Cells were treated with ligand for 24 h. After incubation, cells were processed with RNase A and stained with PI for detection through flow cytometer. The numbers 1, 2 and 3 written on figure represent G_0_/G_1_, S and G_2_/M phases of the cell cycle in HeLa cells, respectively. Data are representative of three identical experiments. (B-D) Effect of NMK-TD-100 on chromosomes of HeLa cells. Cultured HeLa cells were fixed and stained with DAPI (1 μg/mL) for the observation of chromosomes in different stages of mitosis; also, normal and abnormal interphase nuclei are observed in the absence (control cells) and in the presence of NMK-TD-100 (2.5- 5 μM) for 24 h. Arrow indicates mitotic cell. (E) Effect of NMK-TD-100 on mitosis in HeLa cells. Cultured HeLa cells were treated with 0-5 μM NMK-TD-100 for 24 h. Cells were then fixed and stained with DAPI (1 μg/mL). Mitotic indices were determined by counting interphase and mitotic cells at 40X magnification using a confocal microscope. At least 1000 cells per data point were counted. Data are represented as the mean ±SD [*p<0.05 vs. control, where n=3].

Figure 5. Effect of NMK-TD-100 on interphase and spindle microtubules in HeLa cells. (A-I) NMK-TD-100 perturbed the organization of interphase microtubule in HeLa cells. Cells were incubated in various concentration of NMK-TD-100 (0-5 μM) for 24 h, fixed and stained using antibody against α-tubulin (red) and nucleus with DAPI (blue). (J-O) NMK-TD-100 depolymerized the spindle microtubule with formation of multipolar spindles in HeLa cells. Microtubules were stained with antibody against α-tubulin (red) and nucleus with DAPI (blue). Details of the experiments are described in the ‘Materials and methods’ section.

Figure 6. Effect of NMK-TD-100 on reassembly of cold depolymerized microtubule in HeLa cells. Panel A shows normal interphase microtubule in HeLa cells before incubating the cells at 4°C whereas panel B shows microtubule network after 3 h of cold depolymerization. Cold media was replaced by warm media in the presence of 0 (Panel C), 2.5 (Panel D) and 5 μM (Panel E) of NMK-TD-100 and incubated for 3 h at 37°C. Microtubules were tagged by anti- α-tubulin antibody, subsequently TRITC conjugated secondary antibody (red) and nucleus were probed by DAPI (blue). Further details of the experimental procedures are given in the ‘Materials and Methods’ section.

Figure 7. Effect of NMK-TD-100 on tubulin polymerization *in cell-free system*. (A) Tubulin was polymerized in the presence of various concentration of NMK-TD-100 (0-30 μM) and the kinetics of polymerization was monitored by increase using light scattering at 350 nm. (B) Transmission electron microscopic studies of products of tubulin polymerization in the absence and presence of 25 μM of NMK-TD-100. Scale bar is 500 nm. (C) Effect of NMK-TD-100 on depolymerization of preformed microtubule as monitored by decrease in light scattering at 350 nm. The end point absorbance at 350 nm was plotted against the concentration of NMK-TD-100. Data are representative of average of three similar experiments with SD.

Figure 8. Binding of NMK-TD-100 to tubulin as assessed by enhancement of ligand fluorescence. (A) Fluorescence emission spectra of NMK-TD-100 (2 μM) in buffer (curve a) and in the presence of 2 (curve b), 5 (curve c), 7.5 (curve d), 10 (curve e) and 15 (curve f) μM of tubulin. (B) Fluorescence emission spectra of NMK-TD-100 (20 μM) in the presence of aqueous buffer (PEM buffer) and methanol. (C) Scatchard Plot of NMK-TD-100 binding to tubulin. (D) Job Plot for binding of NMK-TD-100 to tubulin. The concentrations of NMK-TD-100 and tubulin were varied continuously whereas the total concentration of tubulin and NMK-TD-100 was kept fixed at 5 μM. The corrected fluorescence value at 450 nm was plotted against the mole fraction of NMK-TD-100. The excitation and emission wavelengths were 340 and 450nm respectively for all the above panels. Data are representative of three similar experiments. Details of all the experiments are described in the ‘Materials and methods’ section.

Figure 9. Characterization of binding of NMK-TD-100 to tubulin as assessed by quenching of fluorescence of tryptophan residues of tubulin. (A) NMK-TD-100 reduced the intrinsic tryptophan fluorescence of tubulin (1 μM) in a concentration-dependent manner. (B) Double reciprocal plot of NMK-TD-100 binding to tubulin. F_max_ was determined from the plot of 1/(F_0_  - F) versus 1/[NMK-TD-100]. (C) Linear plot of NMK-TD-100 binding to tubulin. The excitation and emission wavelengths were 295 and 335 nm, respectively for all the four panels. Data are representative of three identical experiments. (D) The semi-logarithmic plot of ln(Q_max_ -Q_t_) versus time.

Figure 10. Characterization of binding site for NMK-TD-100. (A) Time-dependent binding of NMK-TD-100 to preformed tubulin-colchicine complex as monitored by the fluorescence enhancement of NMK-TD-100. The excitation and emission wavelengths were 390 and 450 nm, respectively. (B) Characterization of concentration-dependent binding of NMK-TD-100 to preformed tubulin-colchicine complex. Tubulin (2 μM) was incubated with varied concentration of colchicine (0-100 μM) to form tubulin-colchicine complexes. 5 μM of NMK-TD-100 was added to each of preformed tubulin-colchicine complex and incubated for 30 mins. The enhancement of NMK-TD-100 fluorescence (at 450 nm) was measured for each sample after excitation at 390 nm. Curves 1, 2, 3, 4, 5 and 6 represent fluorescence emission spectra of NMK-TD-100 in presence of 0, 5,10,25,50 and 100 μM colchicine complexed with tubulin. (C) The emission maxima of NMK-TD-100 at 450 nm in presence of tubulin-colchicine complexes were plotted against different concentrations of colchicine. (D)Vinblastine did not inhibit the binding of NMK-TD-100 to tubulin. The excitation and emission wavelengths were 340 and 450 nm, respectively. Data are representative average of three identical experiments along with SD.

Figure 11. Docking of NMK-TD-100 to tubulin. (A) *In silico* binding of NMK-TD-100 (yellow) on tubulin heterodimers. In the ribbon diagram grey represents α-tubulin monomer and blue represents β-tubulin monomer. (B) 3D diagram representing binding site of NMK-TD-100 on tubulin heterodimers. Red is showing the polar domains, blue stands for non-polar domains and white stands for neutral domains on protein surface. (C) Ball and stick figure showing positions of hydrogen bonds which stabilizes NMK-TD-100 molecule on protein surface. (D) The crystal structure of αβ heterodimer of tubulin depicting the proximity of the binding sites for colchicine (yellow) and NMK-TD-100 (red).

Table 1: Effect of NMK-TD-100 on distribution pattern of HeLa cells in different mitotic phases.

| NMK-TD-100 (µM) | Interphase (% of Cells) | Prophase/  Pro-Metaphase (% of Cells) | Metaphase (% of Cells) | Anaphase/Telophase (% of Cells) |
| --- | --- | --- | --- | --- |
| 0 | 94.44±4.32 | 1.46±0.45 | 2.04±0.37 | 2.33±0.14 |
| 2.5 | 70.62±3.23 | 6.61±1.14 | 22.33±5.21 | 1.32±0.18 |
| 5 | 56.30±2.12 | 8.37±1.92 | 33.75±3.08 | 1.04±0.013 |

Table 2: The amino acid residues which lie within the binding pocket of NMK-TD-100 molecule in tubulin.

| Tubulin  Heterodimer | Secondary  Structures | Amino Acid  Residues |
| --- | --- | --- |
| α | β 1 | GLY 10, GLY 11, ALA 12, GLN 15 |
|  | β 2 | ASP 69, LEU 70, GLU 71 |
|  | β 3 | ALA 99, ASN 101 |
|  | β 4 | SER 140, PHE 141 |
|  | β 5 | GLY 142, GLY 143, GLY144, THR 145, GLY 146, ILE 171,  TYR 172, PRO 173, ALA 174, VAL 177, SER 178, THR 179,  GLU 183. |
|  | β 6 | ASN 206, GLU 207, TYR 224, ASN 228. |
| β | T7 | LEU 248, ASN 249, ALA 250 |
|  | H8 | LYS 254 |

Supporting Information:

Table S1: Binding Parameters:
